# Altered large‐scale individual‐based morphological brain network in spinocerebellar ataxia type 3

**DOI:** 10.1111/cns.14332

**Published:** 2023-06-30

**Authors:** Shu Su, Runhua Sha, Haishan Qiu, Jianping Chu, Liping Lin, Long Qian, Manshi Hu, Chao Wu, Gerald L. Cheung, Zhiyun Yang, Yingqian Chen, Jing Zhao

**Affiliations:** ^1^ Department of Radiology, The First Affiliated Hospital Sun Yat‐sen University Guangzhou China; ^2^ Department of Biomedical Engineering, College of Engineering Peking University Beijing China; ^3^ Department of Neurology, The First Affiliated Hospital Sun Yat‐Sen University Guangzhou China; ^4^ Spin Imaging Technology Co., Ltd Nanjing China

**Keywords:** morphological brain networks, spinocerebellar ataxia type 3, structural MRI, topological organization

## Abstract

**Background:**

Accumulating evidences indicate regional gray matter (GM) morphology atrophy in spinocerebellar ataxia type 3 (SCA3); however, whether large‐scale morphological brain networks (MBNs) undergo widespread reorganization in these patients remains unclear.

**Objective:**

To investigate the topological organization of large‐scale individual‐based MBNs in SCA3 patients.

**Methods:**

The individual‐based MBNs were constructed based on the inter‐regional morphological similarity of GM regions. Graph theoretical analysis was taken to assess GM structural connectivity in 76 symptomatic SCA3, 24 pre‐symptomatic SCA3, and 54 healthy normal controls (NCs). Topological parameters of the resulting graphs and network‐based statistics analysis were compared among symptomatic SCA3, pre‐symptomatic SCA3, and NCs groups. The inner association between network properties and clinical variables was further analyzed.

**Results:**

Compared to NCs and pre‐symptomatic SCA3 patients, symptomatic SCA3 indicated significantly decreased integration and segregation, a shift to “weaker small‐worldness”, characterized by decreased *C*_p_, lower *E*_loc,_ and *E*_glob_ (all *p* < 0.005). Regarding nodal properties, symptomatic SCA3 exhibited significantly decreased nodal profiles in the central executive network (CEN)‐related left inferior frontal gyrus, limbic regions involving the bilateral amygdala, left hippocampus, and bilateral pallidum, thalamus; and increased nodal degree, efficiency in bilateral caudate (all *p*_FDR_ <0.05). Meanwhile, clinical variables were correlated with altered nodal profiles (*p*_FDR_ ≤0.029). SCA3‐related subnetwork was closely interrelated with dorsolateral cortico‐striatal circuitry extending to orbitofrontal‐striatal circuits and dorsal visual systems (lingual gyrus‐striatal).

**Conclusion:**

Symptomatic SCA3 patients undergo an extensive and significant reorganization in large‐scale individual‐based MBNs, probably due to disrupted prefrontal cortico‐striato‐thalamo‐cortical loops, limbic‐striatum circuitry, and enhanced connectivity in the neostriatum. This study highlights the crucial role of abnormal morphological connectivity alterations beyond the pattern of brain atrophy, which might pave the way for therapeutic development in the future.

## Graphical abstract

Symptomatic SCA3 patients undergo an extensive and significant reorganization in large‐scale individual‐based morphological brain networks, probably due to disrupted prefrontal cortico‐striato‐thalamo‐cortical loop, limbic‐striatum circuitry, and enhanced connectivity in the neostriatum.

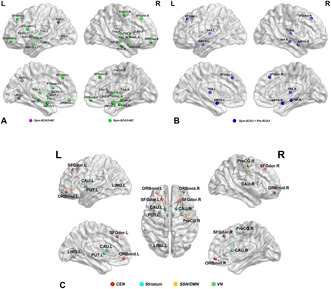

## 1 INTRODUCTION

Spinocerebellar ataxia type 3 (SCA3) is caused by a dynamic cytosine‐adenine‐guanine (CAG) repeat expansion of the Ataxin‐3 gene (*ATXN3*) and is the most common SCA worldwide,^1^ which characterized by the presence of progressive cerebellar ataxia, associated with a wide range of nonataxic symptoms (pyramidal signs, extrapyramidal features, cognitive dysfunction, and dementia). Currently, few therapeutic options show efficacy in the treatment of SCA3. Hence, understanding the morphological abnormalities and large‐scale cerebral network alterations is critical for understanding the pathophysiology and therapeutic development.

Nowadays, structural neuroimaging has shown that SCA3 suffered from infratentorial cerebellar^2^, ^3^ and supratentorial cerebral GM loss/atrophy,^4^, ^5^, ^6^ especially cerebellar GM. To better understand the disease, we previously explored GM volume abnormalities in patients with SCA3 from pre‐symptomatic to symptomatic stage, and widespread atrophic GM volumes were observed in symptomatic stage, including bilateral pallidum, brainstem, pons, and cerebellar,^7^ then pre‐symptomatic stage suffered from white matter damage but no apparent GM loss/atrophy. These results^2^, ^3^, ^4^, ^5^, ^6^, ^7^ provide a large number of evidences for macromorphological abnormalities in SCA3 patients. However, the brain, especially the cerebral hemisphere, is a complex network composed of interconnected regions.^8^, ^9^ Except for the morphological alterations, it is essential to consider the role of regional volume alterations in the context of the whole brain network topology.

Morphological brain networks (MBNs) have become essential for studying human brain connectomes. A cortical feature‐based structural connectivity network can locate atrophied cortical regions and indicate how their connectivity and functions may change. To date, altered GM organization has been reported in SCA3.^10^, ^11^, ^12^ Guo et al.^10^ have constructed brain networks at the group level using a structural covariance approach, which limits the understanding of cerebral loss/atrophy effects at the individual level. Another two studies are based on 1.5 T 3D‐T1 imaging,^11^, ^12^ which might limit the understanding of pathological and biological information about diseases due to insufficient image resolution.^13^ Over recent years, individual‐based MBNs of GM from a large‐scale organization have become increasingly popular,^14^, ^15^, ^16^, ^17^, ^18^, ^19^ depict patterns of interregional relations in regional brain morphology at the individual level, which can provide a more comprehensive understanding of cerebral loss/atrophy pattern into the pathogenesis of SCA3. However, the organization of large‐scale GM MBNs in SCA3 patients from pre‐symptomatic to the symptomatic stage is not fully estimated.

Considering distinct infratentorial cerebellar loss/atrophy affecting the organization of whole brain morphological networks, only supratentorial cerebral GM was taken into constructing the networks in this study. Thus, we compute individual‐based GM networks based on the inter‐regional morphological similarities of GM^20^ in a large sample of SCA3 patients, including pre‐symptomatic (*n* = 24) and symptomatic SCA3 (*n* = 76). We further examine whether the clinical scale for the assessment and rating of ataxia (SARA) scores and disease duration of SCA3 are associated with significant brain GM network properties.

## 2 MATERIALS AND METHODS

### 2.1 Participants and clinical assessment

This study protocol was approved by the ethics committees of our institution and registered at https://clinicaltrials.gov/ (Identifier: ChiCTR2100045857). Written informed consent was obtained from all participants.

**FIGURE 1:**
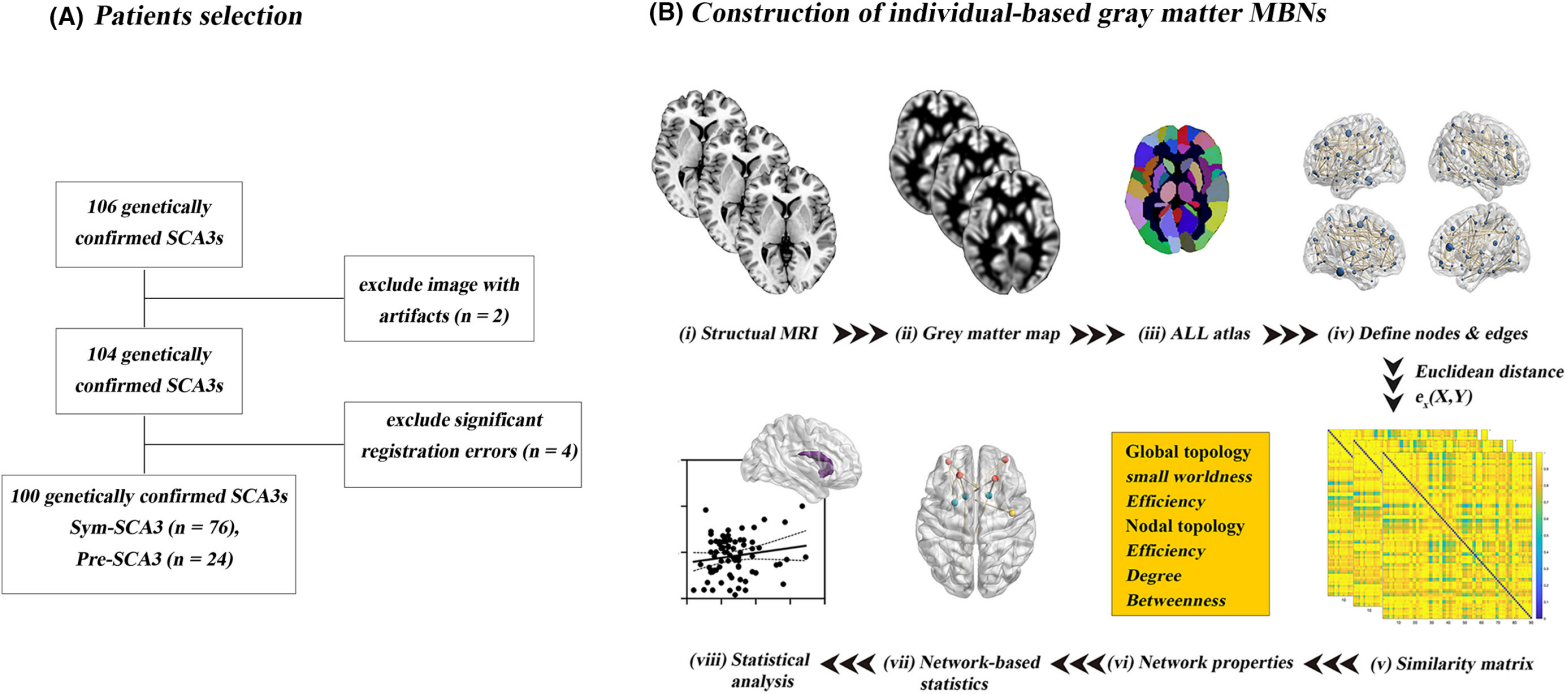
Flowchart of selection of SCA3 patients (A) and the main analytical process of gray matter MBNs (B) in the current study. (i) 3D T1‐weighted imaging and (ii) preprocessing (segment, normalize, modulate, and smooth); (iii) nodes are defined according to the automated anatomical labeling (AAL) atlas; (iv) edges are defined according to the combined Euclidean distance method; (v) an individual similarity matrix is obtained; and (vi, vii) network properties are calculated and analyzed.

From May, 2018 to August, 2021, a total of 106 patients with *ATXN3* mutation carriers were prospectively recruited. The clinical disease severity was assessed via the scale for the assessment and rating of ataxia (SARA) for each patient.^21^ Accordingly, SCA3 patients were grouped as pre‐symptomatic SCA3 (Pre‐SCA3, SARA score <3) and symptomatic SCA3 group (Sym‐SCA3, SARA scores ≥3).^21^ Meanwhile, 54 healthy normal controls (NCs) matched for age, gender, and BMI with an unknown family history of SCA3 were recruited. Detailed clinical and demographic data of the participants are shown in Table 1. A total of 106 SCA3 patients were enrolled, and then six patients were excluded due to image artifacts (*n* = 2) and significant registration errors (*n* = 4) (Figure 1A).

**TABLE 1:** Demographic data and clinical characteristics.

	SCA3 (*n* = 100)	Sym‐SCA3 (*n* = 76)	Pre‐SCA3 (*n* = 24)	NC (*n* = 54)	*p* (SCA3 vs. NC)	*p* (Sym‐SCA3 vs. NC)	*p* (Pre‐SCA3 vs. NC)	*p* (Sym‐SCA3 vs. Pre‐SCA3)
Age, year (Mean ± SD, range)	37.79 ± 11.46 (18–65)	40.72 ± 10.82 (18–65)	28.50 ± 8.09 (18–51)	40.19 ± 11.79 (23–65)	0.222	0.788	<0.001^*^	<0.001^*^
Male/Female (*n*)	49/51	33/43	16/8	24/30	0.589	0.908	0.070	0.047^*^
Course of disease, year (Mean ± SD, range)	4.45 ± 3.94 (0–20)	5.82 ± 3.55 (1–20)	0.13 ± 0.34 (0–1)	NA	NA	NA	NA	<0.001^*^
SARA (Mean ± SD, range)	10.46 ± 8.12 (0–34.5)	13.63 ± 6.68 (4–34.5)	0.42 ± 0.65 (0–2)	NA	NA	NA	NA	<0.001^*^

*Note*: Data are represented as the mean ± SD. For comparisons of demographics, *p*‐values are obtained using two‐sample *t*‐test or Chi‐square test.

^*^ *p* < 0.05 was considered significant.

Abbreviations: NC, normal controls; Pre‐SCA3, pre‐symptomatic spinocerebellar ataxia type 3; SARA, the scale for the assessment and rating of ataxia; SCA3, spinocerebellar ataxia type 3; SD, standard deviation; Sym‐SCA3, symptomatic spinocerebellar ataxia type 3.

The inclusion criteria of SCA3: (1) the molecular genetic testing has proved all the patient's diagnosis; (2) age >18 years; (3) right‐handedness. The exclusion criteria of each participant were as follows: (1) combined with other mental disorders, personality disorders, or psychotropic drug dependence; (2) challenging to cooperate during MRI examination, and the image quality is too poor for image analysis; (3) a history of other brains organic and metabolic diseases; (4) pregnant and lactating women; and (5) other MRI contraindications.

### 2.2 Image acquisition and preprocessing

All participants underwent sagittal three‐dimensional T1 imaging with a 3.0‐T MR imaging system (MAGNETOM Verio scanner; Siemens). Images were acquired using the Magnetization Prepared Rapid Gradient Echo (MPRAGE) sequence with the following parameters: repetition time (TR) = 1750 ms, echo time (TE) = 2.8 ms, 256 sagittal slices with slice thickness = 0.7 mm with no slice gap, a field of view (FOV) = 260 mm × 260 mm^2^, and data matrix = 384 × 384, acquisition time 4 min 21 s.

Automated segmentation of the whole brain based on 3D T1‐weighted images was processed with the CAT12 toolbox (http://www.neuro.uni‐jena.de/cat/) within the SPM12 environment (http://www.fil.ion.ucl.ac.uk/spm/software/spm12/) running under MATLAB R2019b (MathWorks). The preprocessing steps involved spatial normalization of the MNI space and segmentation. Modulated GM images were resliced to a 2‐mm isotropic voxel size and spatially smoothed using a 3D Gaussian kernel with an FWHM of 6 mm.

### 2.3 Construction of individual morphological similarity networks

The Anatomical Automatic Labeling (AAL‐90) atlas was applied^22^ to define network nodes or cerebral regions, and each hemisphere was divided into 45 anatomical regions of interest (ROIs). Next, the approach named Multivariate Euclidean Distances (MEDs)^20^ was performed to estimate the inter‐regional morphological similarities between each of the 4005 pairs of the 90 cortical and subcortical regions derived from each individual GMV Map, after which an individual‐based MBNs (90 × 90) was constructed. The details of MEDs were previously described,^20^ and a flowchart of the construction of individual‐based gray matter MBNs is presented in Figure 1B.

### 2.4 Network analysis

GRETNA toolbox (http://www.nitrc.org/projects/gretna/)^23^ in MATLAB was used to calculate network properties. The minimum and maximum sparsity values were determined to ensure that the thresholder networks were estimable with sparse properties and that the small‐world index was >1.0.^24^ In the current study, the threshold range was 0.05 < *S* < 0.40 with an interval of 0.05. For the brain networks at each sparsity level, the topologic profiles at both global and nodal levels were calculated. Global network profiles included the clustering coefficient (*C*_p_), characteristic path length (*L*_p_), normalized clustering coefficient (*γ*), normalized characteristic path length (*λ*), small‐world parameters (*σ*), global efficiency (*E*_glob_), local efficiency (*E*_loc_), and nodal network topological profiles including nodal efficiency (*E*_*i*_), nodal degree (*D*_*i*_), and nodal betweenness (*B*_*i*_). Considering the network sparsity‐dependent network characteristics, the area under the curve (AUC) of each global profile (*E*_glob_, *E*_loc_, *C*_p_, *L*_p_, *γ*, *λ*, *σ*) and nodal profile (*B*_*i*_, *E*_*i*_, *D*_*i*_) across a range of interested densities were calculated as the summarized scalar for each measure, denoted as $E_{\text{glob}}^{\mathrm{auc}}$, $E_{local}^{\mathrm{auc}}$, $C_{P}^{\mathrm{auc}}$, $L_{P}^{\mathrm{auc}}$, $\gamma^{\mathrm{auc}}$, $\lambda^{\mathrm{auc}}$, $\sigma^{\mathrm{auc}}$, $B_{i}^{\mathrm{auc}}$, $E_{i}^{\mathrm{auc}}$, $D_{i}^{\mathrm{auc}}$, respectively. Finally, BrainNet Viewer toolbox (https://www.nitrc.org/projects/bnv/) was used to present between‐group differences in nodal profiles.

### 2.5 Network‐based statistics analysis

The network‐based statistics (NBS) method (https://www.nitrc.org/projects/nbs/) was used to identify the differences in pairwise connectivity of nodal characteristics between SCA3 and NCs. First, nodes that exhibited significant between‐group differences in at least one of the three nodal centralities (node degree, efficiency, and betweenness) were chosen. Then, a subnetwork connection matrix for each participant was created. Finally, a set of suprathreshold links between connected components were identified using NBS metrics (threshold = 3.2, *p* < 0.05) with the nonparametric permutation approach (5000 permutations).^24^

### 2.6 Statistical analysis

The SPSS v21.0 (IBM Corp.) was used to perform statistical analysis. The Shapiro–Wilk test was used to evaluate the normality of the data for continuous variables. Qualitative variables were compared by Chi‐squared tests, and quantitative variables were compared using two‐tailed independent‐sample *t*‐tests. A *p* < 0.05 was set as statistically significant.

Between‐group comparisons of the AUC of each network metric were evaluated with analysis of covariance (ANCOVA) and post hoc analyses with diagnosis as fixed factors, age, and gender added to the model as covariates, respectively. The Benjamini–Hochberg false discovery rate (BHFDR) correction was performed for multiple comparisons. Then, partial correlation analysis was used to examine relationships between significant network metrics and the disease symptom severity (reflected by SARA scores), as well as the disease duration, controlling for age and gender as confounding variables (*p*_FDR_ < 0.05).

### 2.7 Reproducibility analyses

Similar network analyses were repeated for reproducibility analysis with an additional Harvard Oxford atlas with 112 brain regions (HOA‐112 atlas, Table S1) to evaluate the potential effects of different parcellation schemes.

## 3 RESULTS

### 3.1 Demographic information and clinical characteristics

Finally, 100 SCA3 (49 males; mean age, 37.79 ± 11.46 years) were enrolled, including 76 Sym‐SCA3 (33 males; mean age, 40.72 ± 10.82 years) and 24 pre‐SCA3 (16 males; mean age, 28.50 ± 8.09 years). The Sym‐SCA3 group was older than the Pre‐SCA3 group (*p* < 0.001). Compared to NCs, Pre‐SCA3 presented at a younger age (*p* < 0.001). Detailed demographic and clinical characteristics are summarized in Table 1.

### 3.2 Between‐group differences in global properties of GM networks

In the defined threshold range, Sym‐SCA3, Pre‐SCA3, and NCs exhibited normalized *C*_p_ values (greater than 1) and *L*_p_ values (approximately equal to 1), which showed typical features of small‐world architecture in GM morphological networks. Significant differences in *E*_glob_ (*F* = 10.04, *p* < 0.001), *E*_loc_ (*F* = 23.54, *p* < 0.001), *L*_p_ (*F* = 6.97, *p* = 0.001), *C*_p_ (*F* = 14.16, *p* < 0.001), *γ* (*F* = 7.32, *p* = 0.001), and *λ* (*F* = 6.55, *p* = 0.002) were observed among the Sym‐SCA3, Pre‐SCA3, and NCs groups. However, no significant difference was observed in *λ* (*F* = 1.39, *p* = 0.25) among group comparisons.

**FIGURE 2:**
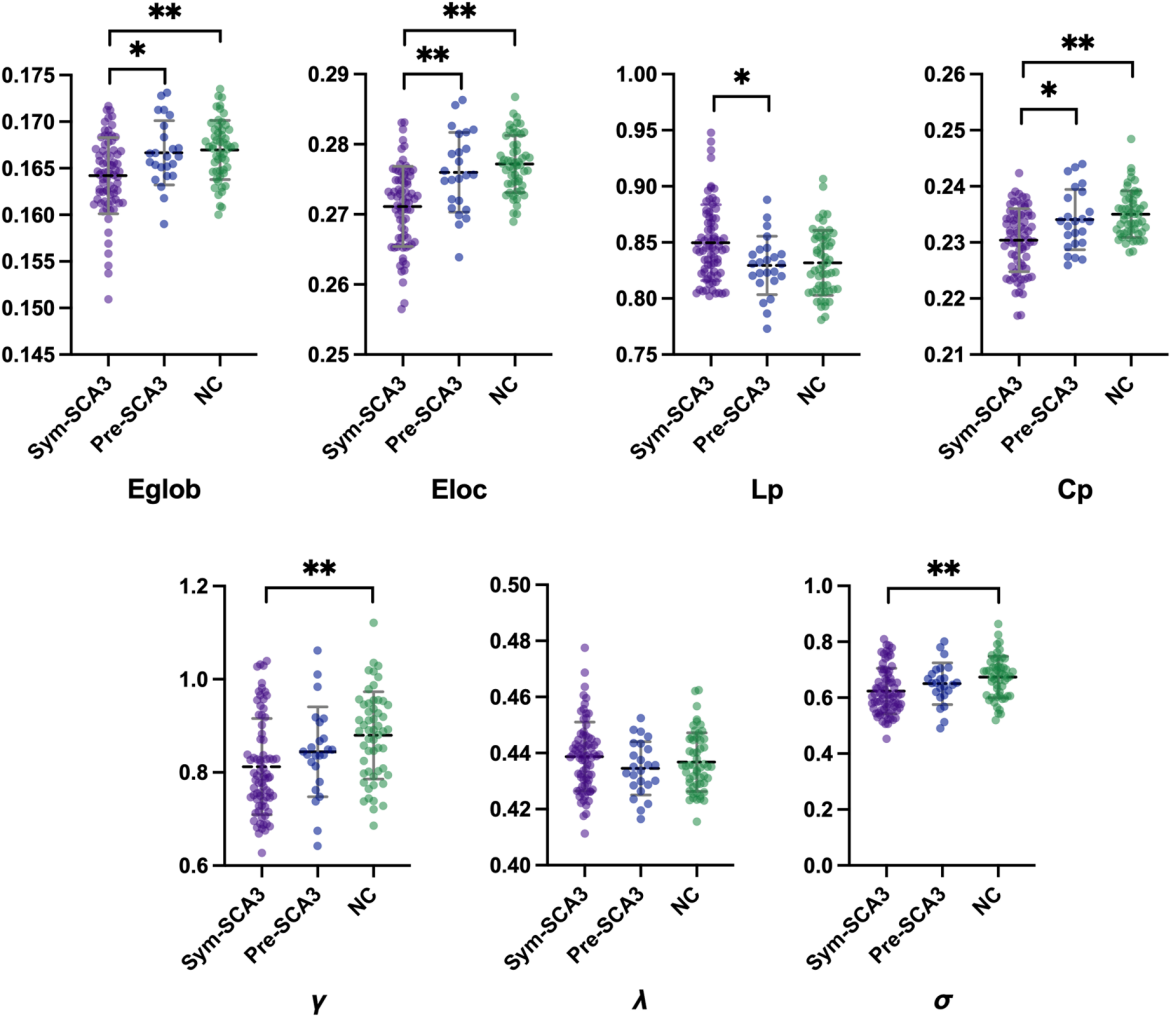
Comparison of global parameters of the brain anatomical networks between the spinocerebellar ataxia type 3 (SCA3) patients and normal controls (NC). *C*_p_, clustering coefficient; *E*_glob_, global efficiency; *E*_loc_, local efficiency; *L*_p_, shortest path length; NC, normal controls; Pre‐SCA3, pre‐symptomatic spinocerebellar ataxia type 3; Sym‐SCA3, symptomatic spinocerebellar ataxia type 3; *γ*, normalized clustering coefficient; *δ* = *λ*/*γ*, small‐world characteristic; *λ*, normalized characteristic path length. Error bars represent the standard deviation of the mean. *p < 0.05, **p < 0.001, compared with NCs.

Post hoc analyses showed that, compared with NCs, Sym‐SCA3 patients exhibited significantly decreased *E*_glob_ (*p* < 0.001), *E*_loc_ (*p* < 0.001), *L*_p_ (*p* = 0.001), *C*_p_ (*p* < 0.001), *γ* (*p* < 0.001), and *λ* (*p* < 0.001) values. Meanwhile, compared to Pre‐SCA3, Sym‐SCA3 exhibited significantly decreased *E*_glob_ (*p* = 0.005), *E*_loc_ (*p* < 0.001), *C*_p_ (*p* = 0.003), and increased *L*_p_ (*p* = 0.006) values (Figure 2). However, no significant differences in global profiles were found between Pre‐SCA3 and NCs groups (Figure 2).

### 3.3 Between‐group differences in nodal profiles of GM networks

**FIGURE 3:**
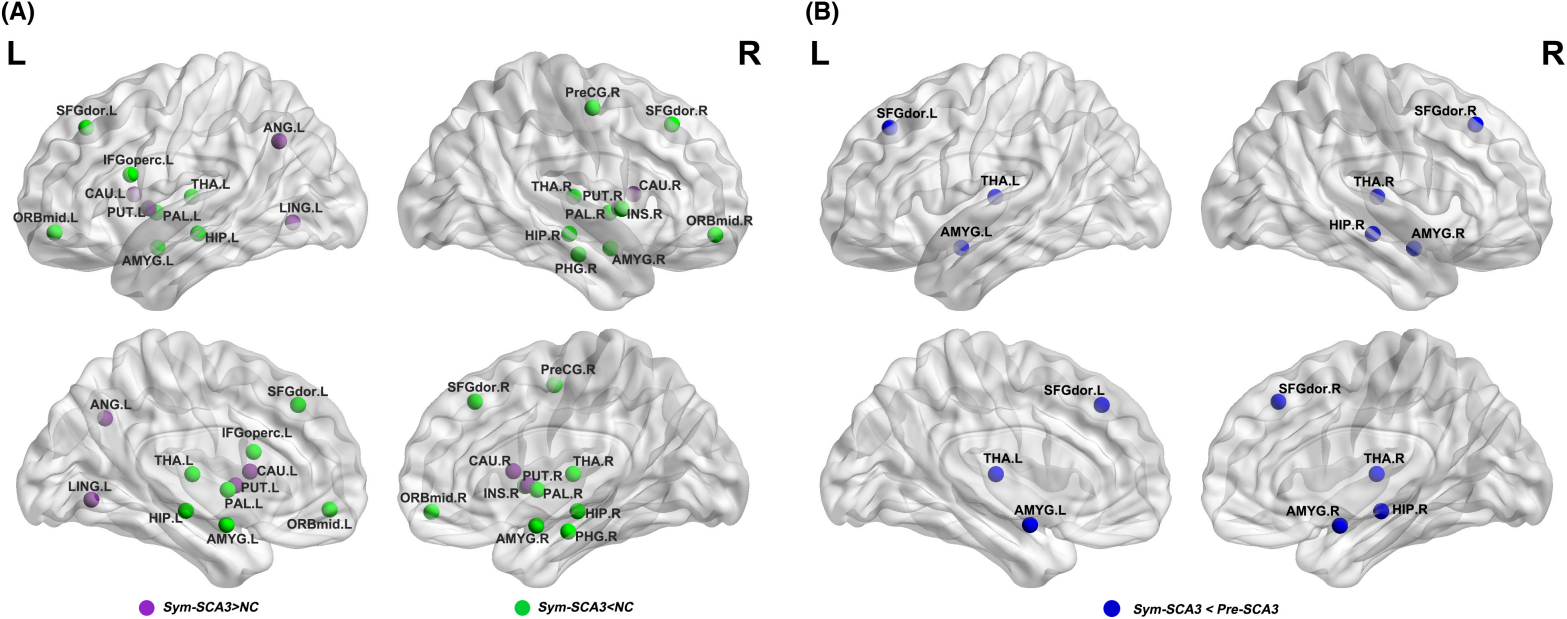
Compared to NCs and Pre‐SCA3 patients, Sym‐SCA3 patients showed regions of altered nodal profiles, showing increased points (purple in A) and decreased (green in A and blue in B) points. The detailed information can be found in Tables 2 and 3. AMYG, amygdala; ANG, angular gyrus; CAU, caudate nucleus; HIP, hippocampus; IFGoperc, inferior frontal gyrus, opercular part; INS, insula; L, left; LING, lingual gyrus; NC, normal controls; ORBmid, middle frontal gyrus, orbital part; PAL, lenticular nucleus, pallidum; PHG, parahippocampal gyrus; PreCG, precentral gyrus; Pre‐SCA3, pre‐symptomatic spinocerebellar ataxia type 3; PUT, lenticular nucleus, putamen; R, right; SFGdor, superior frontal gyrus, dorsolateral; Sym‐SCA3, symptomatic spinocerebellar ataxia type 3; THA, thalamus.

Compared to NCs, Sym‐SCA3 patients showed altered nodal profiles in widespread regions (*p* < 0.05, uncorrected), nine were in the subcortical cortex, six were in the frontal/prefrontal cortex, five were in the temporal cortex, one was in the parietal cortex, and one was in the occipital cortex (Figure 3A, Table 2). Specifically, in comparison with NCs, Sym‐SCA3 exhibited significantly decreased nodal profiles in the left inferior frontal gyrus, opercular part (IFGoperc.L), limbic regions (bilateral amygdala and left hippocampus) and bilateral pallidum, thalamus (*p*_FDR_ < 0.05; Figure 3A, Table 2); as well as significantly increased nodal degree, efficiency in bilateral caudate (*p*_FDR_ <0.05; Figure 3A, Table 2).

**TABLE 2:** Altered nodal profiles in Sym‐SCA3 patients and healthy normal controls (NCs).

Brain regions	Category	*p*‐Value		
		$D_{i}^{\mathrm{auc}}$	$E_{i}^{\mathrm{auc}}$	$B_{i}^{\mathrm{auc}}$
Sym‐SCA3 < NCs				
PreCG.R	SSN/DMN	0.032^a^	0.025^a^	0.099
SFGdor.L	CEN	0.063	0.036^a^	0.191
SFGdor.R	CEN	0.014^a^	0.006^a^	0.576
ORBmid.L	CEN	0.132	0.012^a^	0.095
ORBmid.R	CEN	0.152	0.042^a^	0.102
IFGoperc.L	CEN	0.016^b^	0.003^b^	0.590
HIP.L	Limbic	0.043^b^	0.017^a^	0.492
HIP.R	Limbic	0.010^a^	0.042^a^	0.330
PHG.R	Limbic	0.009^a^	0.009^a^	0.822
AMYG.L	Limbic	0.005^b^	0.005^b^	0.764
AMYG.R	Limbic	0.001^b^	0.004^b^	0.144
INS.R	Limbic	0.016^a^	0.019^a^	0.833
PAL.L	Striatum	0.031^b^	0.002^b^	0.023^b^
PAL.R	Striatum	0.087	0.002^b^	0.089
THA.L	Thalamus	<0.001^b^	<0.001^b^	0.301
THA.R	Thalamus	<0.001^b^	<0.001^b^	0.510
Sym‐SCA3 > NCs				
LING.L	VN	0.015^a^	0.028^a^	0.964
ANG.L	DMN	0.011^a^	0.033^a^	0.532
CAU.L	Striatum	0.008^b^	0.013^b^	0.254
CAU.R	Striatum	0.019^b^	0.032^b^	0.824
PUT.L	Striatum	0.026^a^	0.016^a^	0.044^a^
PUT.R	Striatum	0.216	0.160	0.013^a^

*Note*: Twenty‐two regions with *p*‐value <0.05 in at least one node profile were included.

Abbreviations: AMYG, amygdala; ANG, angular gyrus; $B_{i}^{\mathrm{auc}}$, nodal betweenness; CAU, caudate nucleus; CEN, central executive network; $D_{i}^{\mathrm{auc}}$, nodal degree; $E_{i}^{\mathrm{auc}}$, nodal efficiency; HIP, hippocampus; IFGoperc, inferior frontal gyrus, opercular part; INS, insula; L, left; LING, lingual gyrus; NC, normal controls; ORBmid, middle frontal gyrus, orbital part; PAL, lenticular nucleus, pallidum; PHG, parahippocampal gyrus; PreCG, precentral gyrus; PUT, lenticular nucleus, putamen; R, right; SFGdor, superior frontal gyrus, dorsolateral; SSN, somatosensory network; Sym‐SCA3, symptomatic spinocerebellar ataxia type 3; THA, thalamus; VN, visual network.

^a^ Uncorrected *p* < 0.05.

^b^ *p*_FDR_ < 0.05.

While compared with the Pre‐SCA3 group, Sym‐SCA3 patients showed significantly decreased nodal profiles in the left dorsolateral superior frontal gyrus (SFGdor.L), limbic regions (right hippocampus and left amygdala), and bilateral thalamus (*p*_FDR_ < 0.05; Figure 3B, Table 3).

**TABLE 3:** Decreased nodal profiles in Pre‐SCA3 and Sym‐SCA3 patients.

Brain regions	Category	*p*‐Value		
		$D_{i}^{\mathrm{auc}}$	$E_{i}^{\mathrm{auc}}$	$B_{i}^{\mathrm{auc}}$
Sym‐SCA3 < Pre‐SCA3				
SFGdor.L	CEN	0.004^a^	0.029^b^	0.805
SFGdor.R	CEN	0.029^a^	0.008^a^	0.752
HIP.R	Limbic	0.040^b^	0.004^a^	0.281
AMYG.L	Limbic	0.018^b^	0.028^a^	0.865
AMYG.R	Limbic	0.015^a^	0.055	0.654
THA.L	Thalamus	0.017^b^	0.004^b^	0.052
THA.R	Thalamus	0.017^b^	0.004^b^	0.235

*Note*: Seven regions with *p*‐value <0.05 in at least one node profile were included.

Abbreviations: AMYG, amygdala; $B_{i}^{\mathrm{auc}}$, nodal betweenness; CEN, central executive network; $D_{i}^{\mathrm{auc}}$, nodal degree; $E_{i}^{\mathrm{auc}}$, nodal efficiency; HIP, hippocampus; L, left; Pre‐SCA3, pre‐symptomatic spinocerebellar ataxia type 3; R, right; SFGdor, superior frontal gyrus, dorsolateral; Sym‐SCA3, symptomatic spinocerebellar ataxia type 3; THA, thalamus.

^a^ Uncorrected *p* < 0.05.

^b^ *p*_FDR_ <0.05.

However, no brain regions showed a significant difference in nodal profiles between Pre‐SCA3 and NCs groups.

### 3.4 SCA3‐related subnetwork

**FIGURE 4:**
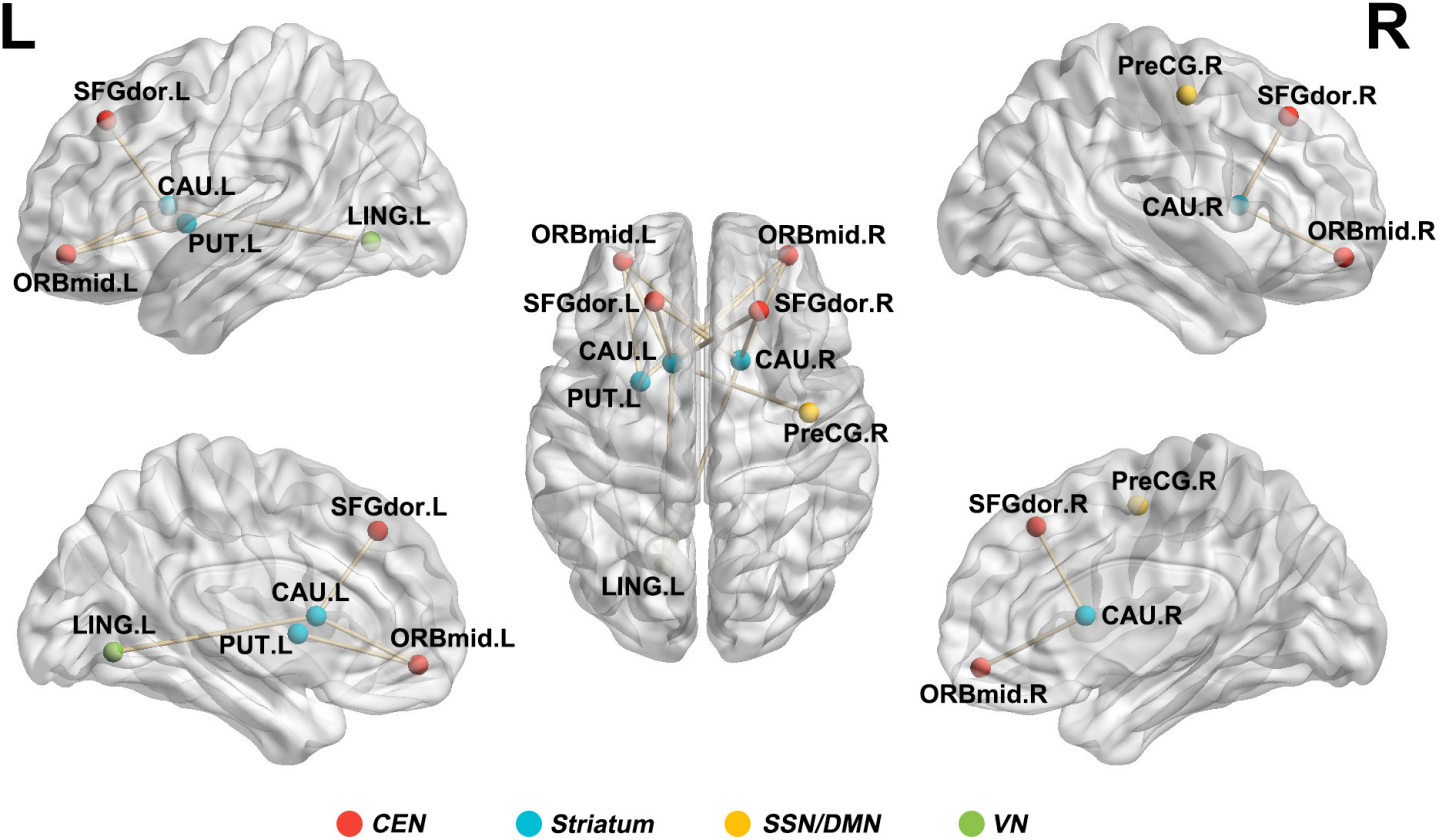
Sym‐SCA3‐related subnetwork. Every node denotes a brain region, and every line represents a connection. Different color nodes represent different brain regions: red, central executive network (CEN); yellow, somatosensory network/default mode network (SSN/DMN); green, visual network (VN); cyan, Striatum. CAU, caudate nucleus; L, left; LING, lingual gyrus; ORBmid, middle frontal gyrus, orbital part; PUT, lenticular nucleus, putamen; R, right; SFGdor, superior frontal gyrus, dorsolateral; THA, thalamus.

For Sym‐SCA3 patients, NBS analysis identified a significantly altered subnetwork with nine nodes and 13 edges (Figure 4). The nodes included components of dorsolateral cortico‐striatal circuitry (dorsolateral superior frontal gyrus, precentral gyrus, caudate, and putamen) extending to orbitofrontal‐striatal circuitry (orbital medial superior frontal gyrus, caudate, and putamen) and dorsal visual systems (lingual gyrus‐striatal). In comparison, no pre‐symptomatic SCA3‐related subnetwork was found by NBS analysis.

### 3.5 Relationships between nodal profiles and clinical variables

**FIGURE 5:**
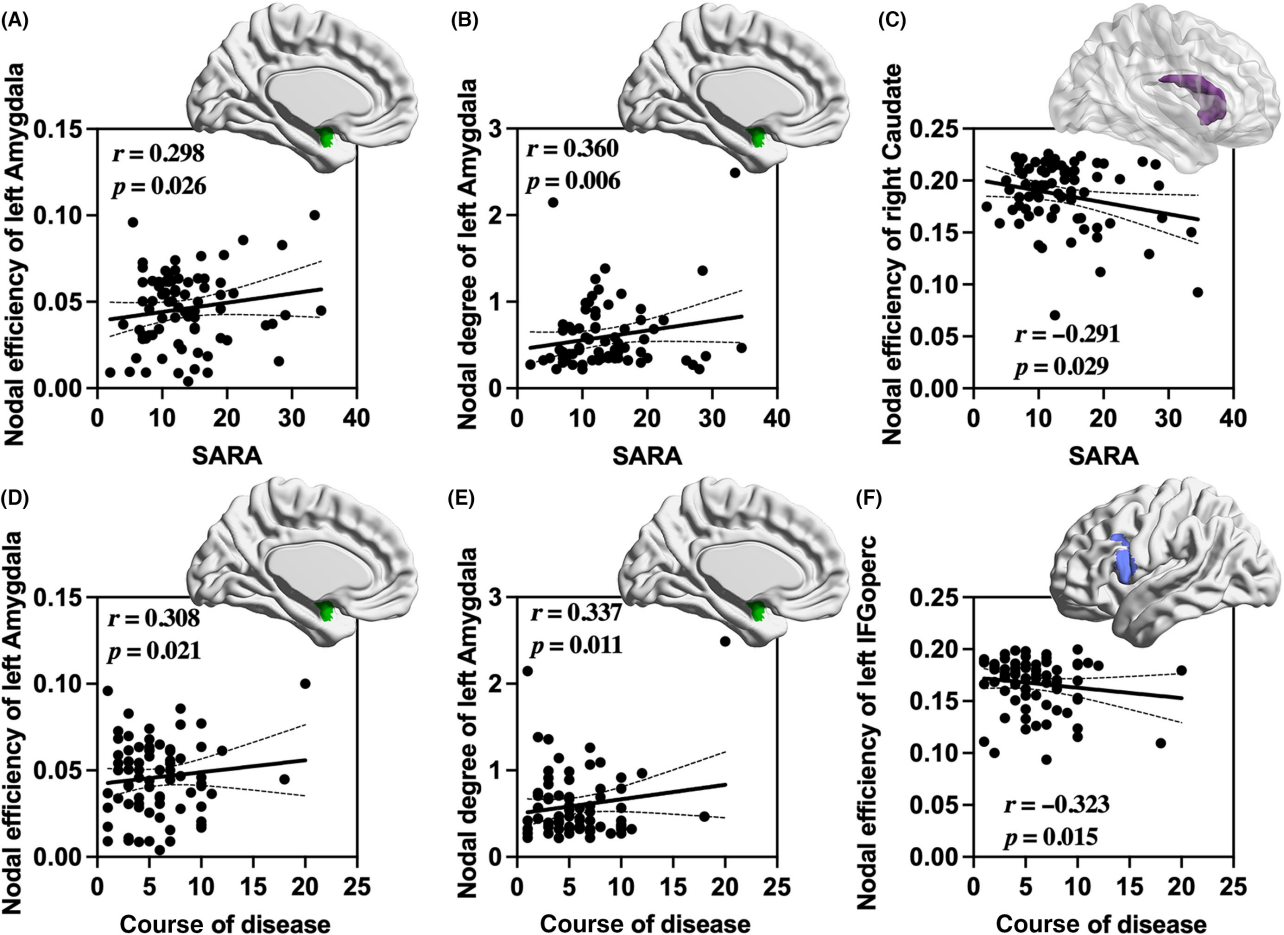
Relationship between the nodal properties and clinical variables in Sym‐SCA3 group. In Sym‐SCA3 patients, SARA scores were positively correlated with the nodal efficiency of the left amygdala (*r* = 0.298; *p*_FDR_ = 0.026, A) and nodal degree of the left amygdala (*r* = 0.360; *p*_FDR_ = 0.006, B). The nodal efficiency of the right caudate (*r* = −0.291; *p*_FDR_ = 0.029, C) was negatively correlated with SARA scores. In addition, the disease duration was positively correlated with the nodal efficiency of the left amygdala (*r* = 0.308; *p*_FDR_ = 0.021, D) and nodal degree of the left amygdala (*r* = 0.337; *p*_FDR_ = 0.011, E). Nodal efficiency of the left inferior frontal gyrus and opercular part (*r* = −0.323; *p*_FDR_ = 0.015, F) were negatively correlated with the disease duration of Sym‐SCA3 patients. SARA, the scale for the assessment and rating of ataxia.

The SARA scores of Sym‐SCA3 patients were positively correlated with nodal efficiency of the left amygdala (Figure 5, *r* = 0.298; *p*_FDR_ = 0.026) and nodal degree of the left amygdala (Figure 5, *r* = 0.360; *p*_FDR_ = 0.006), and negatively correlated with nodal efficiency of right caudate (Figure 5, *r* = −0.291; *p*_FDR_ = 0.029). Besides, in Sym‐SCA3 patients, disease duration was positively correlated with nodal efficiency of the left amygdala (Figure 5, *r* = 0.308; *p*_FDR_ = 0.021) and nodal degree of the left amygdala (Figure 5, *r* = 0.337; *p*_FDR_ = 0.011), and negatively correlated with nodal efficiency of left IFGoperc (Figure 5, *r* = −0.323; *p*_FDR_ = 0.015). No significant correlations were found between clinical variables and any other global or nodal metrics (*p*_FDR_ > 0.05).

### 3.6 Reproducibility of results

We repeated the reconstruction of the GM network using the HOA‐112 atlas. Similar results characterized by lower *E*_glob_ and *C*_p_ were found in Sym‐SCA3 patients than in pre‐SCA3 patients and NCs (Figure S1). In addition, significant altered nodal properties were observed in the prefrontal, limbic, striatum/thalamus in Sym‐SCA3 patients while compared to pre‐SCA3 patients and NCs (details in Tables S2 and S3; Figure S2). Moreover, no significant differences were detected in these network metrics between pre‐SCA3 patients and NCs. Furthermore, Sym‐SCA3 patients had similar disease‐related subnetwork regions, including dorsolateral cortico‐striatal circuitry (dorsolateral superior frontal gyrus), limbic‐striatum circuitry, and dorsal visual systems (fusiform gyrus‐striatal) alterations (Figure S3).

## 4 DISCUSSION

Large‐scale individual‐based MBNs have been performed on Parkinson's disease (PD)^15^, ^16^ and Alzheimer's disease (AD).^17^, ^19^ In comparison, no such analyses have been performed on other ataxias or Wilson's disease so far. The MBN analysis highlights the potential for application to early AD and PD imaging diagnosis. The main findings of our study are as follows: (i) Sym‐SCA3 demonstrated large‐scale disrupted brain network topologies: decreased integration and segregation, while Pre‐SCA3 showed no significant MBN organization alterations; (ii) Sym‐SCA3 showed disrupted prefrontal cortico‐striato‐thalamo‐cortical loops, limbic‐striatum circuitry, and enhanced connectivity in neostriatum; (iii) nodal profiles of left amygdala, right caudate, and left IFGoperc were responsible for symptom severity/duration. Overall, individual network‐level abnormalities extend beyond the pattern of brain atrophy, which provides new insights into the pathophysiology of SCA3.

### 4.1 Decreased integration and segregation at the global level in SCA3

The shorter *C*_p_, lower *E*_loc_, and *E*_glob_ in Sym‐SCA3 indicated decreased integration and segregation of structural brain networks,^8^ consistent with reduced transmission of brain information transmission. Previous structural brain network^11^ showed decreased *C*_p_, *E*_glob_, and increased *L*_p_ (a more sparsity and disrupted structural network) in SCA3. However, a functional connectome study indicated a more regular brain network with higher *C*_p_, *E*_loc_, and modularity in Sym‐SCA3.^25^ Although different networks construction methods (functional connectome,^25^ gray matter MBNs (group‐level structural covariance network^10^ and individual‐level MBNs^11^ and this study)), the results turn out consistent or controversial, which may be due to the continuous remodeling of brain morphological/functional network as the disease progress.

### 4.2 Disrupted prefrontal cortico‐striato‐thalamo‐cortical loops, limbic system, and enhanced connectivity in neostriatum in SCA3

Our work confirms and highlights the disrupted prefrontal cortico‐striato‐thalamo‐cortical loops associated with Sym‐SCA3, which were previously assumed^26^ and partly supported Guo et al.'s research.^10^ The dorsolateral and orbitofrontal prefrontal‐striatal circuits project from the basal ganglia to the prefrontal cortex via the thalamus's ventroanterior and dorsomedial regions.^27^ Disruption in this circuitry demonstrates a ‘fronto‐subcortical’ profile with a pattern of deficits such as impaired set‐shifting and impairment of spatial working memory.

Another vital circuitry involved with SCA3 was the limbic system. The limbic system^28^ is widely connected with other brain structures (neocortex, thalamus, and brain stem), exchanging information among the midbrain, diencephalon, and neocortex. We considered lower connectivity in the limbic system with other brain structures responsible for ataxia symptoms in Sym‐SCA3. Most importantly, compared to pre‐SCA3, the limbic regions may be affected in the initial stage of Sym‐SCA3. Additionally, dorsal visual systems (lingual gyrus‐striatal) were also found in Sym‐SCA3. We speculate that the visual network is participating in compensation into brain network organization for ataxia symptoms.

These findings also extend beyond brain atrophy patterns related to Sym‐SCA3.^7^ The Sym‐SCA3 suffered distinct neostriatum (caudate/putamen) atrophy but with hyper‐connectivity in neostriatum referring to individual‐level MBNs.

### 4.3 Significant relation between nodal network profiles and clinical variables in SCA3

Decreased nodal efficiency and degree of the left amygdala were positively correlated with SARA scores and disease course. Amygdala is a vital part of the limbic system^28^ and is crucial in a wide array of effective and motivation‐related behaviors. Previous functional connectivity networks^25^ reported that bilateral amygdala, putamen was transferred into the sensorimotor network in SCA3 when compared to NCs. Based on current findings, the amygdala may be an important treatment target for SCA3 ataxia symptoms by enhancing internal connectivity in the amygdala.

Increased nodal efficiency of the right caudate was negatively corrected with symptom severity in SCA3, which highlights the compensatory and supportive interactions in neostriatum (caudate/putamen) in Sym‐SCA3. Besides, decreased nodal efficiency of CEN‐related left IFGoperc was negatively corrected with the disease course. Within the progress of the disease, the connectivity involving CEN is persistently damaged, emphasizing the importance of early treatment intervention for SCA3.

### 4.4 Validation analyses

Validation analyses showed that significantly decreased *E*_glob_ and *C*_p_, as well as significantly altered nodal properties in the prefrontal, limbic, striatum/thalamus was similarly observed using the HOA‐112 atlas in Sym‐SCA3 than in NCs. Meanwhile, Sym‐SCA3 showed decreased nodal degree and efficiency in the bilateral thalamus and amygdala according to both the AAL‐90 and HOA‐112 atlas, which uncovered the early involvement of the bilateral thalamus and amygdala. In addition, the SCA3‐related subnetwork was similar to those obtained from the AAL‐90 atlas, implying the robustness of these findings.

### 4.5 Limitations

The current study has two limitations. First, longitudinal studies are needed to examine neurodegenerative alterations from the pre‐SCA3 to Sym‐SCA3 stage to evaluate the impact of the emergence and course of brain atrophy abnormalities on large‐scale brain network organization. Second, due to the distinct infratentorial cerebellar loss/atrophy would affect the organization of whole brain morphological networks, we did not include the infratentorial brain structures in the analysis, which might further limit the exploration of their roles of network alterations in SCA3. Further study with sophisticated network construction methods should be carried out here.

## 5 CONCLUSION

In summary, our findings suggest that Sym‐SCA3 patients undergo an extensive and significant reorganization in MBNs, probably due to disrupted prefrontal cortico‐striato‐thalamo‐cortical loops, limbic‐striatum circuitry, and enhanced connectivity in the neostriatum. Detailed knowledge of large‐scale network reorganization has the potential to help researchers better understand the pathophysiological mechanisms of SCA3 and facilitate the development of therapeutic strategies. In particular, therapeutic modulation of network areas (e.g., limbic‐related amygdala and striatum) by noninvasive or invasive stimulation technologies may improve clinical symptoms.

## AUTHOR CONTRIBUTIONS

Conceptualization and study design: Shu Su, Runhua Sha, Jing Zhao, and Yingqian Chen; Methodology: Shu Su, Runhua Sha, Long Qian, Gerald L. Cheung, Jing Zhao, and Yingqian Chen; Investigation: Shu Su, Runhua Sha, Liping Lin, Haishan Qiu, Manshi Hu, and Chao Wu; Formal Analysis: Shu Su, Runhua Sha, Liping Lin, and Haishan Qiu; Writing—Original Draft: Shu Su, Runhua Sha, and Yingqian Chen; Writing – Review and Editing: Shu Su, Runhua Sha, Zhiyun Yang, Long Qian, Jianping Chu, Manshi Hu, Chao Wu, Yingqian Chen, and Jing Zhao; Visualization: Shu Su and Runhua Sha. All the coauthors listed above gave their final approval of this manuscript version.

## FUNDING INFORMATION

This study was supported by the Guangdong Basic and Applied Basic Research Foundation, China (No. 2021A1515012279) and Science, Technology Program of Guangzhou, China (202201011244, 202201011228), the Natural Science Fund Youth Science Fund Project of China (No. 82001439, No. 82172015), and the Medical Scientific Research Foundation of Guangdong Province (No. A2020327). The authors of this manuscript declare no relationships with any companies whose products or services may be related to the article's subject matter.

## CONFLICT OF INTEREST STATEMENT

The authors of this manuscript declare no relationships with any companies whose products or services may be related to the article's subject matter.

## CONSENT TO PARTICIPATE

Written informed consent was obtained from all subjects.

## Supporting information


Appendix S1

## Data Availability

Data generated or analyzed during the study are available from the corresponding author by request. The data are not publicly available due to privacy or ethical restrictions.
