# Supplementary material for: Altered large‐scale individual‐based morphological brain network in spinocerebellar ataxia type 3
Source: CNS Neurosci Ther. 2023 Jun 30;29(12):4102–12. doi: 10.1111/cns.14332 (PMC10651944; doi:10.1111/cns.14332)
Supplement: Supplementary file 1 — Appendix S1 [file CNS-29-4102-s001.docx]

**Supplementary Materials**

**Table S1.** Cortical and subcortical regions of HOA-112 Atlas.

| Index | Region | Abbr. | Index | Region | Abbr. |
| --- | --- | --- | --- | --- | --- |
| (1,2) | Frontal Pole | FP | (57,58) | Cingulate Gyrus, anterior division | CGa |
| (3,4) | Insular Cortex | INS | (59,60) | Cingulate Gyrus, posterior division | CGp |
| (5,6) | Superior Frontal Gyrus | F1 | (61,62) | Precuneous Cortex | PCN |
| (7,8) | Middle Frontal Gyrus | F2 | (63,64) | Cuneal Cortex | CN |
| (9,10) | Inferior Frontal Gyrus, pars triangularis | F3t | (65,66) | Frontal Orbital Cortex | FOC |
| (11,12) | Inferior Frontal Gyrus, pars opercularis | F3o | (67,68) | Parahippocampal Gyrus, anterior division | PHa |
| (13,14) | Precentral Gyrus | PRG | (69,70) | Parahippocampal Gyrus, posterior division | PHp |
| (15,16) | Temporal Pole | TP | (71,72) | Lingual Gyrus | LG |
| (17,18) | Superior Temporal Gyrus, anterior division | T1a | (73,74) | Temporal Fusiform Cortex, anterior division | TFa |
| (19,20) | Superior Temporal Gyrus, posterior division | T1p | (75,76) | Temporal Fusiform Cortex, posterior division | TFp |
| (21,22) | Middle Temporal Gyrus, anterior division | T2a | (77,78) | Temporal Occipital Fusiform Cortex | TOF |
| (23,24) | Middle Temporal Gyrus, posterior division | T2p | (79,80) | Occipital Fusiform Gyrus | OF |
| (25,26) | Middle Temporal Gyrus, temporooccipital part | TO2 | (81,82) | Frontal Operculum Cortex | FO |
| (27,28) | Inferior Temporal Gyrus, anterior division | T3a | (83,84) | Central Opercular Cortex | CO |
| (29,30) | Inferior Temporal Gyrus, posterior division | T3p | (85,86) | Parietal Operculum Cortex | PO |
| (31,32) | Inferior Temporal Gyrus, temporooccipital part | TO3 | (87,88) | Planum Polare | PP |
| (33,34) | Postcentral Gyrus | POG | (89,90) | Heschl's Gyrus (includes H1 and H2) | H |
| (35,36) | Superior Parietal Lobule | SPL | (91,92) | Planum Temporale | PT |
| (37,38) | Supramarginal Gyrus, anterior division | SGa | (93,94) | Supracalcarine Cortex | SCLC |
| (39,40) | Supramarginal Gyrus, posterior division | SGp | (95,96) | Occipital Pole | OP |
| (41,42) | Angular Gyrus | AG | (97,98) | bed nucleus of the stria terminalis central division | Bst |
| (43,44) | Lateral Occipital Cortex, superior division | OLs | (99,100) | Thalamus | Thal |
| (45,46) | Lateral Occipital Cortex, inferior division | OLi | (101,102) | Caudate | Caud |
| (47,48) | Intracalcarine Cortex | CALC | (103,104) | Putamen | Put |
| (49,50) | Frontal Medial Cortex | FMC | (105,106) | Pallidum | Pall |
| (51,52) | Juxtapositional Lobule Cortex (formerly Supplementary Motor Cortex) | SMC | (107,108) | Hippocampus | Hip |
| (53,54) | Subcallosal Cortex | SC | (109,110) | Amygdala | Amy |
| (55,56) | Paracingulate Gyrus | PAC | (111,112) | Accumbens | Accbns |

Note: The regions are listed according to a prior template obtained from an HOA atlas; the odd number represents the corresponding brain regions in the left hemisphere; the even number denotes the specific brain regions in the right hemisphere.

**Table S2.** Altered nodal profiles in SCA3 patients and healthy normal controls (NCs).

| **Brain regions** | **Category** | ***P*-value** | | |
| --- | --- | --- | --- | --- |
|  |  | $D_{i}^{auc}$ | $E_{i}^{auc}$ | $B_{i}^{auc}$ |
| **Sym-SCA3 < NCs** | | | | |
| F1.L | CEN | 0.039* | 0.010* | 0.120 |
| INS.R | Limbic | 0.047** | 0.019* | 0.020 |
| CGa.L | DMN | 0.012* | 0.079 | 0.028* |
| CGa.R | DMN | 0.048** | 0.024** | 0.197 |
| Thal.L | Thalamus | 0.008** | 0.019** | 0.645 |
| Thal.R | Thalamus | 0.004** | 0.019** | 0.697 |
| Amy.L | Limbic | <0.001** | <0.001** | 0.300 |
| Amy.R | Limbic | <0.001** | <0.001** | 0.492 |
| **Sym-SCA3 > NCs** | | | | |
| TFa.L | VN | 0.005* | 0.008* | 0.757 |
| TFp.L | VN | 0.023** | 0.022** | 0.139 |
| TOF.L | VN | 0.018* | 0.034** | 0.458 |
| Caud.L | Striatum | 0.023** | 0.022** | 0.970 |
| Caud.R | Striatum | 0.006* | 0.005* | 0.022* |
| Put.L | Striatum | 0.008* | 0.006* | 0.998 |
| Put.R | Striatum | 0.129 | 0.047* | 0.148 |
| Accbns.L | Striatum | 0.043* | 0.036* | 0.164 |
| Accbns.R | Striatum | 0.030* | 0.023* | 0.019* |

**Note:** 17 regions with *P*-value <0.05 in at least one node profile were included. * Uncorrected *P* < 0.05; ***P_FDR_* < 0.05. Abbreviations: $D_{i}^{auc}$= nodal degree; $E_{i}^{auc}$= nodal efficiency; $B_{i}^{auc}$= nodal betweenness; Sym-SCA3 =symptomatic spinocerebellar ataxias type 3; NC = normal controls; F1 = Superior Frontal Gyrus; INS = Insular Cortex; CGa = Cingulate Gyrus, anterior division; Thal = Thalamus; Amy = Amygdala; TFa = Temporal Fusiform Cortex, anterior division; TFp = Temporal Fusiform Cortex, posterior division; TOF = Temporal Occipital Fusiform Cortex; Caud = Caudate; Put = Putamen; Accbns = Accumbens; L = left; R = right; CEN = central executive network; DMN = default network; VN = visual network.

**Table S3.** Decreased nodal profiles in Pre-SCA3 and Sym-SCA3 patients.

| **Brain regions** | **Category** | ***P*-value** | | |
| --- | --- | --- | --- | --- |
|  |  | $D_{i}^{auc}$ | $E_{i}^{auc}$ | $B_{i}^{auc}$ |
| **Sym-SCA3 < Pre-SCA3** | | | | |
| Thal.L | Thalamus | 0.038* | 0.153 | 0.699 |
| Thal.R | Thalamus | 0.008* | 0.026* | 0.448 |
| Amy.L | Limbic | 0.002* | 0.017* | 0.467 |
| Amy.R | Limbic | 0.044* | 0.028* | 0.752 |
| **Sym-SCA3 > Pre-SCA3** | | | | |
| Caud.L | Striatum | 0.031* | 0.061 | 0.713 |
| Put.L | Striatum | 0.018* | 0.034* | 0.292 |
| Accbns.L | Striatum | 0.044* | 0.028* | 0.752 |

Note: 7 regions with *P*-value <0.05 in at least one node profile were included. * Uncorrected P < 0.05. ** *P_FDR_* < 0.05. Abbreviations: $D_{i}^{auc}$= nodal degree; $E_{i}^{auc}$= nodal efficiency; $B_{i}^{auc}$= nodal betweenness; Sym-SCA3 =symptomatic spinocerebellar ataxias type 3; Pre-SCA3 = pre-symptomatic spinocerebellar ataxias type 3; Thal = Thalamus; Amy = Amygdala; Caud = Caudate; Put = Putamen; Accbns = Accumbens; L = left; R = right.

**Figure Legends**


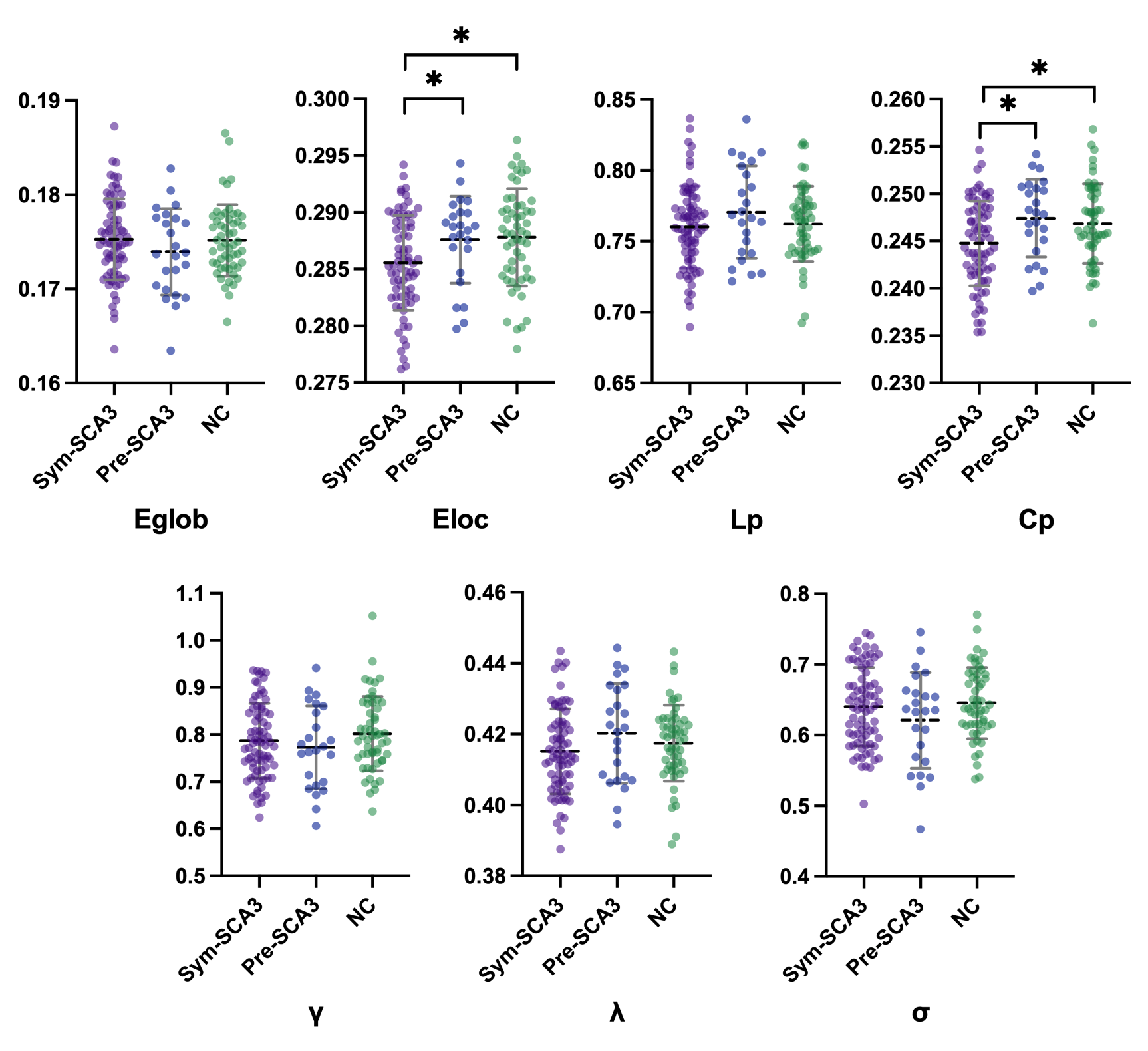


**Figure S1. Comparison of global parameters of the brain anatomical networks between the spinocerebellar ataxias type 3 (SCA3) patients and normal controls (NC).** Abbreviations: Sym-SCA3, symptomatic spinocerebellar ataxias type 3; Pre-SCA3, pre-symptomatic spinocerebellar ataxias type 3; NC, normal controls; Eglob, global efficiency; Eloc, local efficiency; Cp, clustering coefficient; Lp, shortest path length; λ, normalized characteristic path length; γ, normalized clustering coefficient; δ = λ/γ, small-world characteristic. Error bars represent the standard deviation of the mean. **P* < 0.05, compared with NCs.


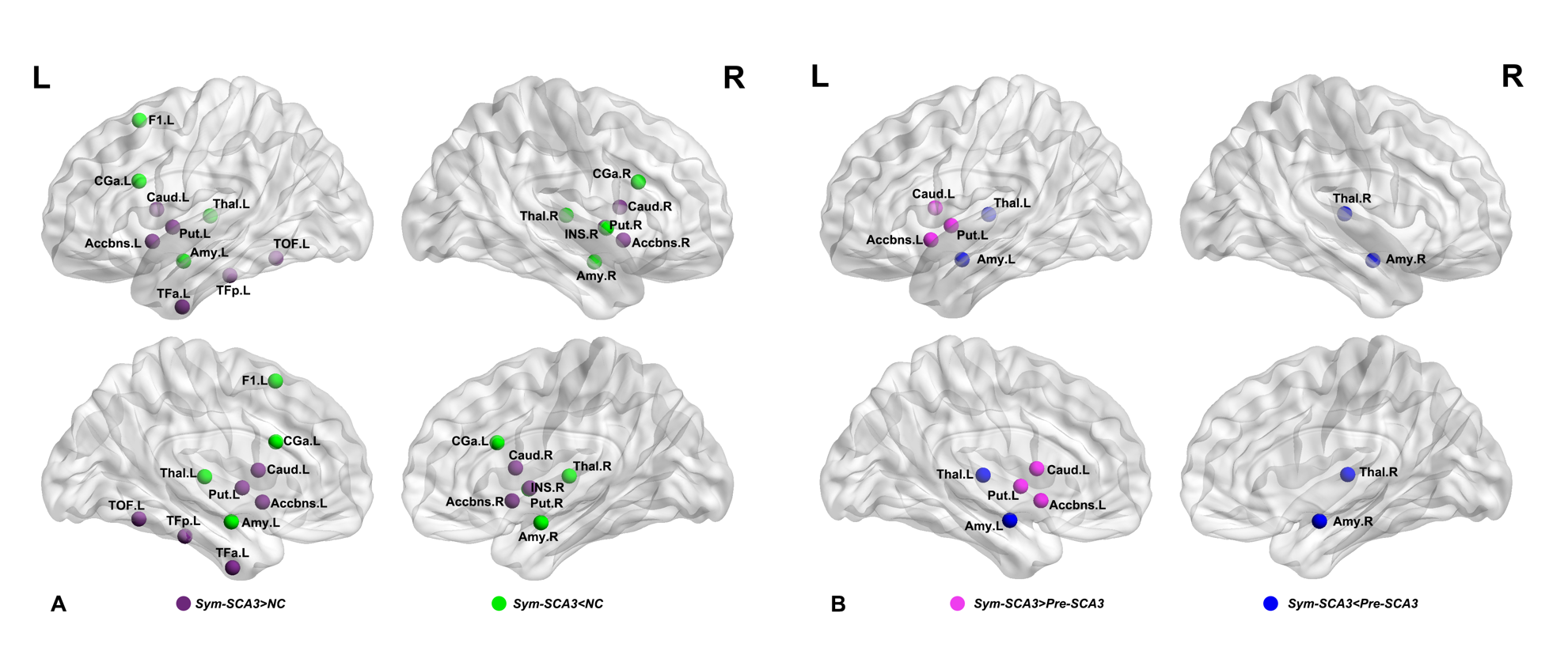


**Figure S2. Compared to NCs and Pre-SCA3 patients, Sym-SCA3 patients showed regions of altered nodal profiles, showing increased points (purple in A) and decreased (green in A & blue in B) points.** The detailed information can be found in **Table S2&3**. Abbreviations: Sym-SCA3 =symptomatic spinocerebellar ataxias type 3; Pre-SCA3 = pre-symptomatic spinocerebellar ataxias type 3; NC = normal controls; F1 = Superior Frontal Gyrus; INS = Insular Cortex; CGa = Cingulate Gyrus, anterior division; Thal = Thalamus; Amy = Amygdala; TFa = Temporal Fusiform Cortex, anterior division; TFp = Temporal Fusiform Cortex, posterior division; TOF = Temporal Occipital Fusiform Cortex; Caud = Caudate; Put = Putamen; Accbns = Accumbens; L = left; R = right.


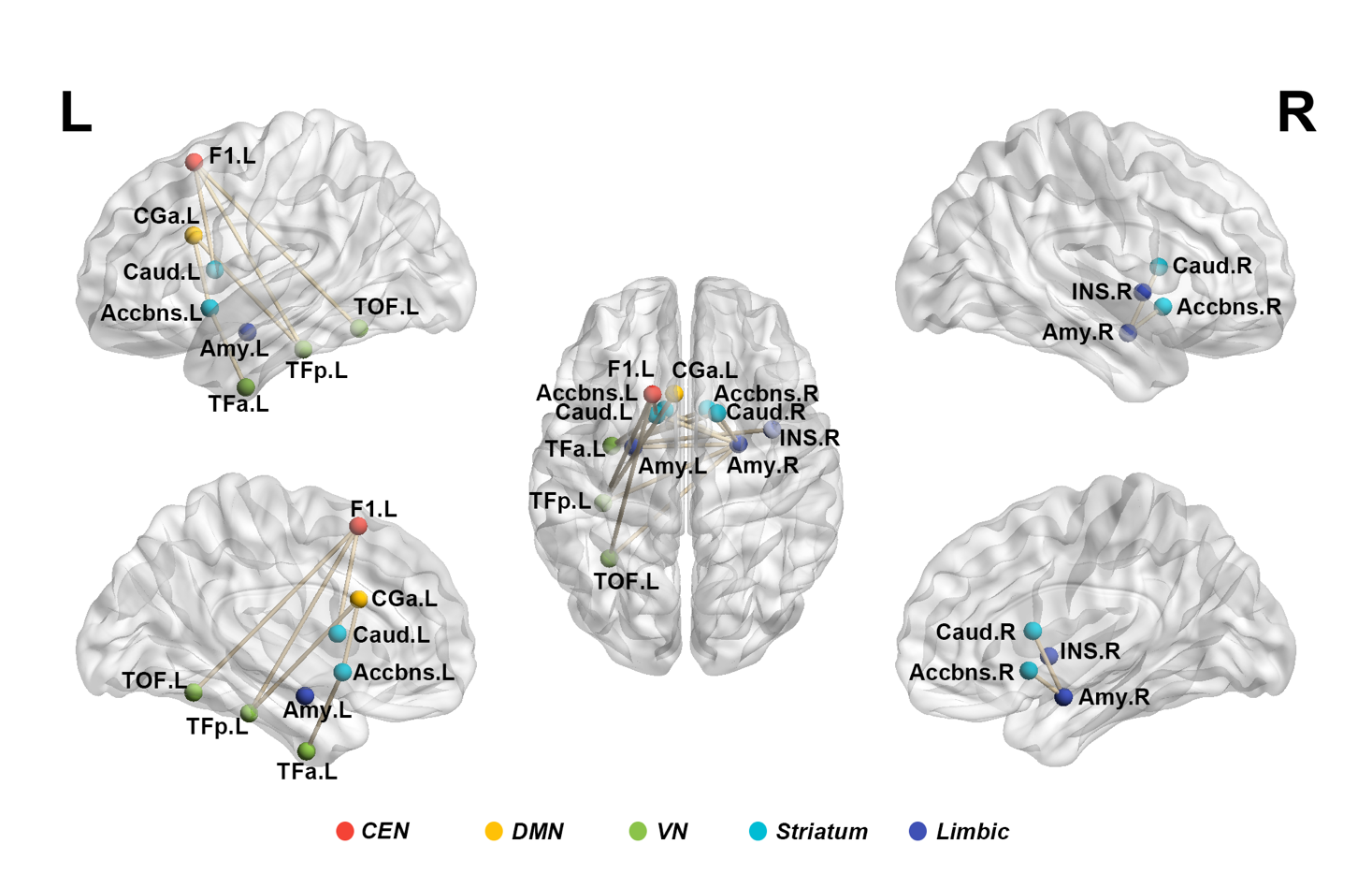


**Figure S3**. **Sym-SCA3-related subnetwork.** Every node denotes a brain region, and every line represents a connection. Different-color nodes represent different brain regions: red, central executive network (CEN); yellow, somatosensory network/default mode network (DMN); green, visual network (VN); cyan, Striatum; blue, Limbic. F1 = Superior Frontal Gyrus; INS = Insular Cortex; CGa = Cingulate Gyrus, anterior division; Amy = Amygdala; TFa = Temporal Fusiform Cortex, anterior division; TFp = Temporal Fusiform Cortex, posterior division; TOF = Temporal Occipital Fusiform Cortex; Caud = Caudate; Accbns = Accumbens; L = left; R = right.
